# Elucidating the binding mechanism of SARS-CoV-2 NSP6-TBK1 and structure-based designing of phytocompounds inhibitors for instigating the host immune response

**DOI:** 10.3389/fchem.2023.1346796

**Published:** 2024-01-16

**Authors:** Muhammad Suleman, Iqra Ishaq, Haji Khan, Safir Ullah khan, Rehana Masood, Norah A. Albekairi, Abdulrahman Alshammari, Sergio Crovella

**Affiliations:** ^1^ Laboratory of Animal Research Center (LARC), Qatar University, Doha, Qatar; ^2^ Center for Biotechnology and Microbiology, University of Swat, Swat, Pakistan; ^3^ Hefei National Laboratory for Physical Sciences at the Microscale, School of Life Sciences and Medicine, University of Science and Technology of China, Hefei, Anhui, China; ^4^ Department of Biochemistry, Shaheed Benazir Bhutto Women University, Peshawar, Pakistan; ^5^ Department of Pharmacology and Toxicology, College of Pharmacy, King Saud University, Riyadh, Saudi Arabia

**Keywords:** NSP6, TBK1, SARS-CoV-2, molecular docking, MD simulation

## Abstract

SARS-CoV-2, also referred to as severe acute respiratory syndrome coronavirus 2, is the virus responsible for causing COVID-19, an infectious disease that emerged in Wuhan, China, in December 2019. Among its crucial functions, NSP6 plays a vital role in evading the human immune system by directly interacting with a receptor called TANK-binding kinase (TBK1), leading to the suppression of IFNβ production. Consequently, in the present study we used the structural and biophysical approaches to analyze the effect of newly emerged mutations on the binding of NSP6 and TBK1. Among the identified mutations, four (F35G, L37F, L125F, and I162T) were found to significantly destabilize the structure of NSP6. Furthermore, the molecular docking analysis highlighted that the mutant NSP6 displayed its highest binding affinity with TBK1, exhibiting docking scores of −1436.2 for the wildtype and −1723.2, −1788.6, −1510.2, and −1551.7 for the F35G, L37F, L125F, and I162T mutants, respectively. This suggests the potential for an enhanced immune system evasion capability of NSP6. Particularly, the F35G mutation exhibited the strongest binding affinity, supported by a calculated binding free energy of −172.19 kcal/mol. To disrupt the binding between NSP6 and TBK1, we conducted virtual drug screening to develop a novel inhibitor derived from natural products. From this screening, we identified the top 5 hit compounds as the most promising candidates with a docking score of −6.59 kcal/mol, −6.52 kcal/mol, −6.32 kcal/mol, −6.22 kcal/mol, and −6.21 kcal/mol. The molecular dynamic simulation of top 3 hits further verified the dynamic stability of drugs-NSP6 complexes. In conclusion, this study provides valuable insight into the higher infectivity of the SARS-CoV-2 new variants and a strong rationale for the development of novel drugs against NSP6.

## Introduction

In the 21st century, the frequent emergence of coronaviruses, specifically within the Orthocoronavirinae family, has had devastating global consequences (Zhu N. et al., 2020; Perlman, 2020). The current pandemic caused by SARS-CoV-2, which originated in Wuhan, has profoundly impacted societies and economies worldwide (Zhu Z. et al., 2020). COVID-19, the disease caused by SARS-CoV-2, has become a multi-wave pandemic, affecting people with a wide range of symptoms, from mild to severe respiratory illnesses, such as sore throat, fever, headache, dry cough, and breathing difficulties. In severe cases, the virus can significantly impair lung function and even lead to fatalities (Wu and McGoogan, 2020; Zhou et al., 2020). Recently, there have been reports of new strains of the virus in various regions of the world, which are showing increased transmissibility and virulence (Gorbalenya et al., 2020; Harrison et al., 2020; Suleman et al., 2023a; Sayaf et al., 2023). These emerging variants are more infectious, spread more easily, and cause more severe illness. The rapid global spread of SARS-CoV-2 and the emergence of these new variants pose a significant risk to public health. As a result, scientists worldwide are exploring various strategies to combat SARS-CoV-2, such as utilizing integrated multi-omics technologies to develop innovative and potent vaccines and medications (Gu et al., 2021; Li et al., 2023; Panahi et al., 2023).

SARS-CoV-2 possesses a genome of approximately 29.9 kb in length and contains at least 14 open reading frames (ORFs) responsible for encoding various viral proteins (Wu et al., 2020). Within this genome, there are 4 structural proteins, 7 accessory proteins, and 16 non-structural proteins (Xia et al., 2020). Notably, two large overlapping ORFs, namely, ORF1a and ORF1b, are located in the 5-proximal two-thirds of the genome. These ORFs encode continuous polypeptides known as pp1a and pp1ab, which undergo cleavage by viral proteases to produce the 16 non-structural proteins (nsp1-16), collectively forming the replicase (Sundar et al., 2021). The non-structural proteins are mainly associated with viral replication while the structural proteins are accountable factors of infection and also responsible for the virion assembly (Chen et al., 2020; Sundar et al., 2021). The accessory proteins are linked with viral pathogenesis and infection (Xia et al., 2020). NSP6 is classified as one of the non-structural proteins, with a genome size of approximately 34 kDa, and it is characterized by a highly conserved C-terminus and an eight transmembrane helicase structure (Giovanetti et al., 2020). NSP6 along with the NSP3 and NSP4 is accountable for the formation of replicase organelles or replication-transcription complexes, which hold significant importance in the virus’s life cycle and its ability to cause infections (He et al., 2016; Oudshoorn et al., 2016).

The initial defense against viral infections, including coronaviruses, relies on the innate immune system’s production of interferons (IFNs). This response is triggered when specific patterns found in pathogens, called pathogen-associated molecular patterns (PAMPs), such as viral mRNA, are recognized. Key sensors, RIG-I and MDA5, are responsible for detecting these PAMPs (Acharya et al., 2020; Li et al., 2020; Park and Iwasaki, 2020). Once activated, RIG-I and MDA5 bind to the CARD domain of the mitochondrial antiviral signaling protein (MAVs). This activation leads to the recruitment of downstream signaling components, including IKKε and TBK1, to the mitochondria. These downstream components then phosphorylate IRF3 and IRF7, which form dimers and translocate to the nucleus. Inside the nucleus, IRF3 and IRF7 initiate the expression of IFN-I genes (Larson et al., 2003; Kawai and Akira, 2007; Liu et al., 2015; Rashid et al., 2021). IFN-I induction triggers antiviral activity within cells, inhibiting viral replication. One mechanism by which IFN-I accomplishes this is by stimulating the activity of dsRNA-activated kinase. This well-coordinated immune response plays a pivotal role in protecting host cells from viral infections (Balachandran et al., 2000; Malathi et al., 2007). Corona-virus developed diverse strategies to counteract the IFN pathway and to antagonize IFN response by targeting distinct steps in the IFN production pathway (Rajsbaum and García-Sastre, 2013). Earlier investigations have established that NSP6 can hinder the production of IFN-b (Xia et al., 2020; Shemesh et al., 2021). To pinpoint the specific step in the production of IFN-b that NSP6 affects, scientists studied various components of the RIG-1 pathway. The results indicated that when the IFN-b promoter was induced by IKKε, TBK1, or MAVS, the luciferase activity was suppressed. This suggests that NSP6 may inhibit IFN-b production by targeting IRF3 or other component(s) situated upstream of IRF3 in the signaling pathway. Additionally, the study demonstrated that NSP6 can influence the phosphorylation of TBK1 and IRF3. When NSP6 was overexpressed and followed by poly (I:C) transfection, IRF3 phosphorylation was reduced by approximately 57%, but TBK1 phosphorylation remained unaffected. This indicates that NSP6 likely binds to TBK1, leading to decreased IRF3 phosphorylation, which ultimately results in a reduction of the IFN-b production (Xia et al., 2020; Rashid et al., 2022).

The role of NSP6 has been revealed in various studies which inhibit the induction of interferon-beta through interacting with Tank Binding Kinase 1 and escape the immune system (Guo et al., 2021; Vazquez et al., 2021). Considering the importance of NSP6 in human immune evasion, the present study was carried out to investigate the effect of newly emerged and deleterious mutations on NSP6-TBK1 bonding network and evasion of human immune system. Furthermore, we used the virtual drugs screening against the binding interface of NSP6 and TBK1 to halt the binding, thereby controlling the evasion of the human immune system mediated by NSP6. The molecular dynamics simulation approach was further used to verify the stability of top hit drugs and NSP6 complexes.

## Materials and methods

### Sequence retrieval and analysis

The protein sequence of SARS-CoV-2 NSP6 (ID: P0DTD1) and the crystal structure of TBK1 (ID: Q9UHD2) were obtained from the UniProt database (https://www.uniprot.org/) (Consortium, 2019). GISAID (Global Initiative on Sharing All Influenza Data) is a database and platform designed for sharing and analyzing genomic data of influenza viruses and other emerging infectious diseases. To identify single nucleotide substitutions in the NSP6 protein, we submitted the sequence in FASTA format to the GISAID database (https://www.gisaid.org/). By comparing the submitted sequence with the reference sequence hCoV-19/Wuhan/WIV04/2019 (accession no MN996528.1), the server detected new mutations and provided information on the positions of the amino acid residues that were replaced (Kalia et al., 2021).

### Molecular modeling and structural validation

The protein sequence of NSP6 was used as input for the 3D structure modeling process using AlphaFold 2.0. AlphaFold 2.0 is an advanced protein folding prediction system developed by DeepMind, an artificial intelligence research lab owned by Alphabet Inc. It employs deep learning techniques to predict the 3D structure of proteins based on their amino acid sequences (Jumper et al., 2021). We performed further validations using ProSA-web (https://prosa.services.came.sbg.ac.at/prosa.php) and PROCHECK (https://saves.mbi.ucla.edu/). These validations involved analyzing the Ramachandran plot to ensure proper distribution of residues and bond angles (Laskowski et al., 1993; Gopalakrishnan et al, 2007). After constructing the structure, it underwent validation and minimization procedures before further processing.

### Mutational impact on structure stability

Protein structure stability is of paramount importance for various biological processes and functions. Proteins are fundamental building blocks of living organisms, and their structure directly influences their ability to perform specific tasks within cells and organisms. Therefore, to analyze the effect of identified mutations on the structure stability of NSP6, we used the I-Mutant 2.0 serve (https://folding.biofold.org/i-mutant/i-mutant2.0.html). The server needs wild type and mutant protein residue position for determining the impact of substitution of amino acid on protein. A positive value of ∆∆G show high stability and negative value shows less stability (Calabrese et al., 2009). However, for structure-based protein stability, the DynaMut2 server (http://biosig.unimelb.edu.au/dynamut2) was used for finding the effect of alteration in dynamics and protein stability through the Normal Mode Analysis approach. The ΔΔG value less than zero indicates destabilization while a value higher than zero indicates stabilization (Rodrigues et al., 2021). Finally, the destabilizing mutations shortlisted by the aforementioned two servers were subjected to the mCSM server to determine the effect of mutants on protein stability by using a graph-based signature (http://biosig.unimelb.edu.au/mcsm/stability). By analyzing the RSA (Relative Solvent Accessibility) and ΔΔG (difference in free energy) values for each mutation, the mCSM gained valuable insights into how these mutations affected the stability of the proteins under investigation (Pires et al., 2014).

### Mutants generation and variation analysis

The chimera software, a molecular graphics and modeling program developed by the University of California, San Francisco was used to insert the newly identified highly destabilizing mutations in the wildtype structure of NSP6 protein (Webb and Sali, 2016). Furthermore, the mutant structures of NSP6 were subjected to a minimization process which lower the total energy of a protein structure. Following the stability analysis, we used the PyMOL software to compare the structural differences between the wildtype and NSP6 and its variants. We achieved this by superimposing each mutant onto the WT NSP6 structure and then calculating the RMSD (root mean square deviation) value. This allowed us to quantify and understand the extent of structural variations introduced by the mutations in comparison to the original protein structure.

### Bonding network and its free energy calculation

The ClusPro server (https://cluspro.org) is a widely utilized tool for conducting protein-protein docking. Its user-friendly interface only requires two files in Protein Data Bank (PDB) format to initiate the docking process (Kozakov et al., 2017). In this study, we employed the ClusPro server to perform molecular docking between WT NSP6 and various mutants with the TBK1 protein. The server provides the ten best complex models ranked by low energy score as the output of the docking simulation. To visualize the binding interface in terms of hydrogen bond, salt bridge, and non-bonded contacts between the NSP6 and TBK1 complex, we used the PDBsum online server (http://www.ebi.ac.uk/thornton-srv/databases/pdbsum/Generate.html) (Mohammad et al., 2021). Furthermore, we used the MM/GBSA approach to assess the binding free energies of both wild-type and mutant NSP6 complexes. This method is renowned for its reliability in estimating binding free energies for diverse biological complexes (Rashid et al., 2022). The computation of binding free energies was conducted using the MMGBSA. py script, which considers various factors, including electrostatic interactions, van der Waals forces, solvent-accessible surface area (SA), and the generalized Born model (GB).

The binding free energies calculated mathematically by the following equation:

$$“\Delta G\left(bind\right)=\Delta G\left(complex\right)-\;\left[\Delta G\left(receptor\right)+\Delta G\left(ligand\right)\right]$$

To obtain the individual contributions to the total free energy, we used the following equation:

$${\prime \prime G=G}_{bond}{+G}_{ele}{+G}_{vdW\;}{+G}_{pol}{+G}_{npol^{\prime \prime }}$$

### Targeting of NSP6-TBK1 binding interface by virtual drug screening

ANPDB (African Natural Products Databases) is an accumulation of medicinally important natural compounds, therefore, the South African natural product database was downloaded in a 3D structure data file (3D-SDF) from the ANPDB website (http://african-compounds.org/anpdb/). Initially, a computational screening of this database was performed using the FAF-Drugs 4 web-server to identify non-toxic drug-like molecules that adhere to Lipinski’s rule of 5 (Lagorce et al., 2017). Lipinski’s Rule of 5 predicts drug-likeness based on molecular properties: a molecule with no more than 5 hydrogen bond donors, 10 hydrogen bond acceptors, a molecular weight under 500, and a partition coefficient log *p* less than 5 is more likely to have favorable pharmacokinetic properties and oral bioavailability, increasing its potential as a successful drug candidate. The filtered database was then screened against the binding interface of NSP6 and TBK1. For this screening, AUTODOCK Vina 2.0 was employed, initially using 16 exhaustiveness settings for rapid screening. The top hits from this step were selected, and a more detailed screening was carried out using 64 exhaustiveness settings to eliminate false positive results. Next, the top 10% of drugs were selected for induced fit docking (IFD) by using the AutoDockFR (Ravindranath et al., 2015), AutoDockFR typically employs force fields like AMBER or CHARMM, simulation protocols such as molecular dynamics (MD), and scoring functions like AMBER scoring or force-field-based scoring for Induced Fit Docking (IFD) simulations. We used the default parameters for the IFD docking. This technique accommodates receptor flexibility and facilitates a covalent docking. After this process, the MD (Molecular Dynamics) simulations were conducted on the final top 3 hits.

### Molecular dynamics simulation

The Amber20 package was employed for conducting molecular dynamics (MD) simulations, focusing on assessing the stability of the complexes formed between top hit drugs and NSP6. The simulations used the antechamber force field for parameterizing the molecules (Maier et al., 2015). The solvation of each system was achieved using the TIP3P model, and to neutralize the systems, To neutralize the system charge Na+ and Cl + ions were inoculated (Price and Brooks, 2004). The MD simulations were conducted in multiple stages, starting with energy minimization to optimize the initial structures. The energy minimization protocol consisted of 9,000 cycles, with the first 6,000 cycles utilizing the steepest descent minimization (Meza, 2010), followed by the remaining 3,000 cycles using the conjugate gradient minimization (Watowich et al., 1988). This step aimed to eliminate any unfavorable clashes within the system after neutralization. Subsequently, the systems were heated to a temperature of 300K and then equilibrated at constant pressure (1 atm) to achieve a stable starting point for the production phase of the simulation. The production step was then run for a duration of 100ns to gather data for analysis. To accurately account for long-range electrostatic interactions, the particle mesh Ewald method was employed (Petersen, 1995; Salomon-Ferrer et al., 2013). Additionally, the SHAKE algorithm was used to treat covalent bonds, ensuring efficient treatment of these interactions during the simulations (Kräutler et al. 2000). The post-simulation analysis was performed by using the CPPTRAJ and PTRAJ packages of amber20. These packages were utilized to examine the dynamic stability, residual fluctuation, compactness, and hydrogen bonding network of the complexes (Roe and Cheatham, 2013). To assess the structural dynamic stability, the Root Mean Square Deviation (RMSD) was computed. However, the Rg (radius of gyration) was employed to calculate the structural compactness. On the other hand, to analyze the fluctuation at the residues level we calculated the Root Mean Square Fluctuation (RMSF).

## Results and Discussion

**FIGURE 1:**
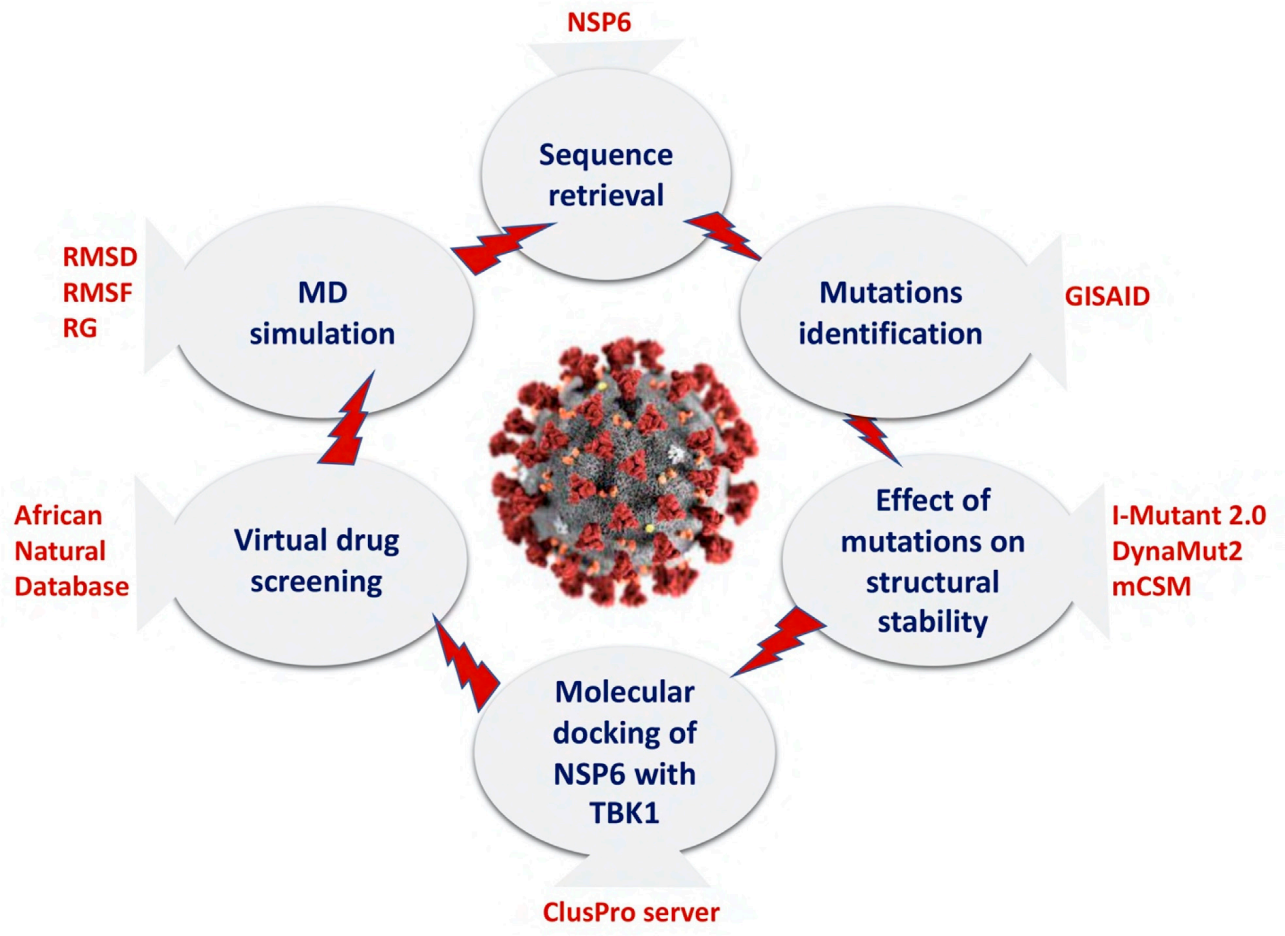
A comprehensive workflow of the current study.

The global pandemic caused by the SARS-CoV-2 genome has become a major cause for concern across the world. Significant efforts are underway to identify a potential molecular drug to combat the virus (Martin and Cheng, 2022). Recently, multiple regions have reported the presence of novel strains of the virus which are demonstrating heightened transmissibility and virulence (Gorbalenya et al., 2020; Harrison et al., 2020; Suleman et al., 2023a; Sayaf et al., 2023). These emerging variants exhibit increased infectivity, spread more readily, and lead to more severe illness. The rapid worldwide dissemination of SARS-CoV-2 and the appearance of these new variants present a substantial threat to public health. In several studies, the function of NSP6, a non-structural protein of SARS-CoV-2, has been revealed to hinder the induction of IFNβ by interacting with Tank Binding Kinase 1 (TBK1), allowing the virus to evade the human immune system (Guo et al., 2021; Vazquez et al., 2021). Recognizing the significance of NSP6 in immune evasion, our current research aimed to examine the impact of newly emerged and harmful mutations on the NSP6-TBK1 binding network and its implications for escaping the human immune response. Additionally, we conducted virtual drug screening targeting the binding interface of NSP6 and TBK1 to disrupt the interaction and potentially control the evasion of the human immune system facilitated by NSP6. Finally, we use the molecular dynamics simulation approach to confirm the stability of identified drugs and NSP6 complexes. Figure 1 illustrates the comprehensive workflow of the current study.

### Sequence retrieval and mutations identification in NSP6

**FIGURE 2:**
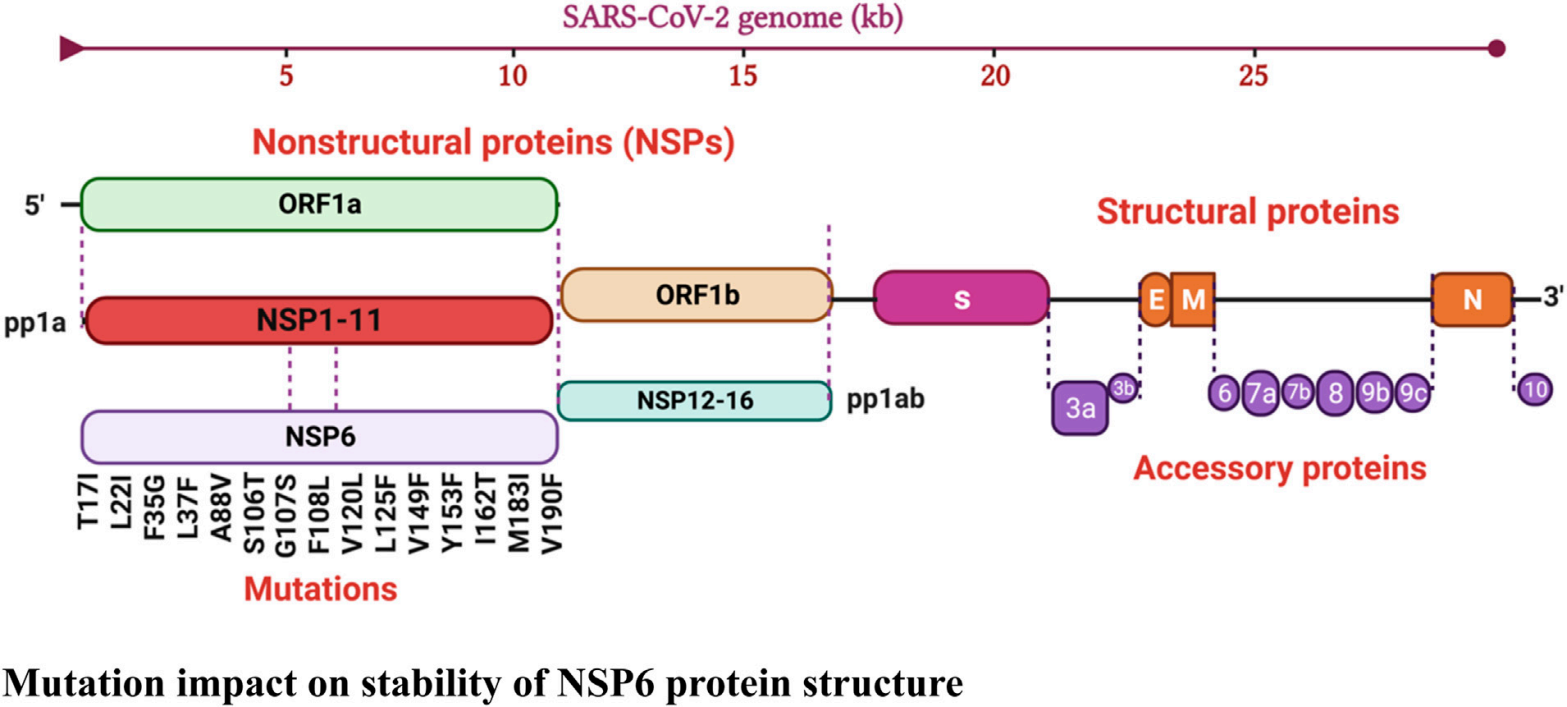
Schematic representation of mutations identified in NSP6 protein.

The protein sequence of SARS-CoV-2 NSP6 (ID: P0DTD1) and the crystal structure of TBK1 (ID: Q9UHD2) were obtained from the UniProt database (https://www.uniprot.org/) (Magrane, 2011; Consortium, 2019). Mutation plays a crucial role in determining the pathogenicity of viruses as it directly affects their capacity to cause disease in a host organism. Compared to other organisms, viruses exhibit notably high mutation rates, leading to rapid evolution, which is a key factor in shaping their ability to cause disease (Suleman et al., 2023b; Aziz et al., 2023). To identify the newly emerged mutations in the NSP6 protein, the retrieved sequence was submitted to the GISAID database which is designed for sharing and analyzing genomic data of influenza viruses and other emerging infectious diseases. The GISAID database identified 15 new mutations (T17I, L22I, F35G, L37F, A88V, S106T, G107S, F108L, V120L, L125F, V149F, Y153F, I162T, M183I, V190F) in the NSP6 protein by comparing the submitted sequence with the reference sequence hCoV-19/Wuhan/WIV04/2019 (Kalia et al., 2021). Figure 2 illustrates the graphical representation of identified mutations.

### Mutation impact on stability of NSP6 protein structure

Certain mutations have the potential to substitute a stabilizing amino acid with a less stable one, causing the protein’s natural structure to be disrupted. Consequently, the protein may become less compact and more susceptible to unfolding or misfolding. On the other hand, there are mutations that can enhance the interactions between amino acids, leading to improved stability compared to the wild-type version of the protein. These beneficial mutations often result in enhanced folding and thermodynamic stability of the protein (Mou et al., 2021; L et al., 2020). Various online servers such as I-Mutant 2.0, DynaMut 2, and mCSM were used to predict the effect of identified mutations on the structural stability of NSP6 protein. First, the identified 15 mutations were submitted to the I-mutant2.0 server to evaluate the structural stability of NSP6. I-Mutant2.0 is a tool that uses a support vector machine (SVM) to predict how single point mutations can impact the stability of proteins. The tool was trained and validated using data from ProTherm, the most extensive database of experimental information on thermodynamic changes in protein stability resulting from mutations under various conditions (Capriotti et al., 2005; Suleman et al., 2023a). After analyzing 15 mutations in the NSP6 protein, I-Mutant2.0 predicted changes in the ∆∆G values ranging from 0.56 kcal/mol to −4.61 kcal/mol. Among these mutations, 14 of them (T17I, F35G, L37F, A88V, S106T, G107S, F108L, V120L, L125F, V149F, Y153F, I162T, M183I, and VI90F) were associated with decreased stability. However, one mutant (L22I) exhibited increased stability. Furthermore, to verify the results generated by I-Mutant2.0 server, we submitted the aforementioned mutations to the DynaMut2 server. DynaMut2 sever utilizes a combination of NMA (Normal Mode Analysis) and graph-based representations of wildtype environment to investigate how single and multiple point mutations affect the stability and dynamics of proteins (Rodrigues et al., 2021). Analysis of 15 variants through DynaMut2 server determined the ∆∆G value ranging from 0.81 kcal/mol to −1.69 kcal/mol, whereas 14 mutations (L22I, F35G, L37F, A88V, S106T, G107S, F108L, V120L, L125F, V149F, Y153F, I162T, M183I and VI90F) decreased structural stability while 1 mutation (T17I) increased structural stability (Table1).

**TABLE 1:** List of deleterious mutations prediction by I-Mutant and DynaMut servers.

Index	Variants	I-Mutant2.0		DynaMut2	
		Predicted∆∆G	Outcome	Predicted∆∆G	Outcome
1	T17I	−0.07 kcal/mol	Destabilizing	0.81 kcal/mol	Stabilizing
2	L22I	0.56 kcal/mol	Stabilizing	−0.29 kcal/mol	Destabilizing
3	F35G	−3.89 kcal/mol	Destabilizing	−1.82 kcal/mol	Destabilizing
4	L37F	−0.05 kcal/mol	Destabilizing	−0.81 kcal/mol	Destabilizing
5	A88V	−0.58 kcal/mol	Destabilizing	−0.55 kcal/mol	Destabilizing
6	S106T	−0.61 kcal/mol	Destabilizing	−0.07 kcal/mol	Destabilizing
7	G107S	−0.84 kcal/mol	Destabilizing	−0.06 kcal/mol	Destabilizing
8	F108L	−3.31 kcal/mol	Destabilizing	−0.11 kcal/mol	Destabilizing
9	V120L	−1.36 kcal/mol	Destabilizing	−0.05 kcal/mol	Destabilizing
10	L125F	−1.18 kcal/mol	Destabilizing	−0.94 kcal/mol	Destabilizing
11	V149F	−3.68 kcal/mol	Destabilizing	−0.65 kcal/mol	Destabilizing
12	Y153F	−0.22 kcal/mol	Destabilizing	−0.33 kcal/mol	Destabilizing
13	I162T	−4.61 kcal/mol	Destabilizing	−1.69 kcal/mol	Destabilizing
14	M183I	−1.64 kcal/mol	Destabilizing	−0.32 kcal/mol	Destabilizing
15	V190F	−2.1 kcal/mol	Destabilizing	−0.83 kcal/mol	Destabilizing

Afterward, to shortlist the destabilized mutations identified by the I-Mutant 2.0 and DynaMut 2 servers we used the mCSM server. The mCSM server presents an innovative method that utilizes graph-based signatures for investigating missense mutations. These signatures encode atomic distance patterns, enabling the representation of the protein residue environment and facilitating the training of predictive models (Pires et al., 2014; Pires et al., 2016; Rodrigues et al., 2019). The top four highly destabilizing mutations revealed by the mCSM server were F35G, L37F, L125F, and I162T with ∆∆G values of −1.928 kcal/mol, −1.214 kcal/mol, −1.207 kcal/mol, and −1.476 kcal/mol respectively (Table 2). Similar approaches were used by several previous studies for the selection of highly destabilizing mutations (Suleman et al., 2021a; Suleman et al., 2022a). To delve deeper into the significance of these mutations within the immune evasion pathway, we processed it for further analysis.

**TABLE 2:** List of deleterious mutations prediction by mCSM server.

Index	Variants	∆∆G mCSM	Outcome
**1**	F35G	−1.928 kcal/mol	**Destabilizing**
**2**	L37F	−1.214 kcal/mol	**Destabilizing**
3	A88V	−0.449 kcal/mol	Destabilizing
4	S106T	−0.365 kcal/mol	Destabilizing
5	G107S	−0.598 kcal/mol	Destabilizing
6	F108L	−0.822 kcal/mol	Destabilizing
7	V120L	−0.326 kcal/mol	Destabilizing
**8**	L125F	−1.207 kcal/mol	**Destabilizing**
9	V149F	−0.794 kcal/mol	Destabilizing
10	Y153F	−0.529 kcal/mol	Destabilizing
**11**	I162T	−1.476 kcal/mol	**Destabilizing**
12	M183I	−0.597 kcal/mol	Destabilizing
13	V190F	−0.962 kcal/mol	Destabilizing

### NSP6 protein modeling and RMSD analysis by superimposition

**FIGURE 3:**
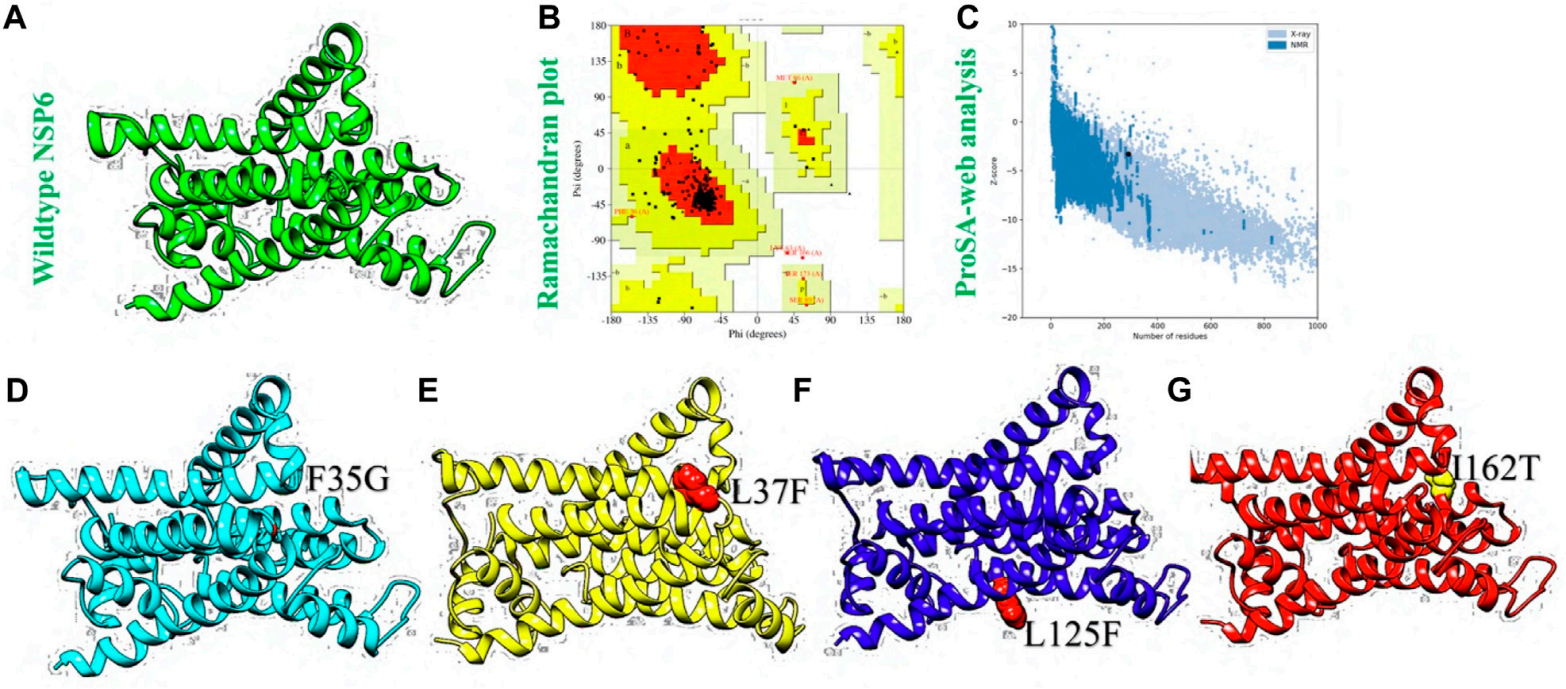
Structural modeling and variants generation of NSP6 protein. **(A)** Represents the AlphaFold generated wildtype NSP6, **(B)** represents Ramachandran plot analysis. **(C)** Represents ProSA-web analysis **(D)** showing F35G protein model, **(E)** showing L37F protein model, **(F)** showing L125F protein model. **(G)** Showing I162T protein model.

The 3D structure of a protein is of paramount importance for its function, as it directly influences how the protein interacts with other molecules and performs its specific biological tasks. Proteins are essential macromolecules in living organisms, and their functions are highly dependent on their unique 3D shapes. Consequently, to model the 3D structure of NSP6, we used the AlphaFold 2.0 which employs deep learning techniques to predict the 3D structure of proteins based on their amino acid sequences (Jumper et al., 2021). The 3D structure of NSP6 protein is shown in Figure 3A. To verify the predicted NSP6 protein model, we used the Ramachandran and ProSA-web analysis. The Ramachandran analysis indicated that 84.3% of the amino acid residues were situated in the most favored regions, with 15% found in additional allowed regions, and only 0.7% located in the disallowed regions (Figure 3B). Furthermore, the ProSA-web analysis yielded a Z score of −3.29 for the predicted NSP6 protein structure, a value well within the expected range for normal protein structures of similar size (Suleman et al., 2022b) (Figure 3C). Finally, the shortlisted destabilizing mutations (F35G, L37F, L125F and I162T) were modeled in the wildtype structure of NSP6 by using the Chimera software (Figures 3D–G).

**FIGURE 4:**
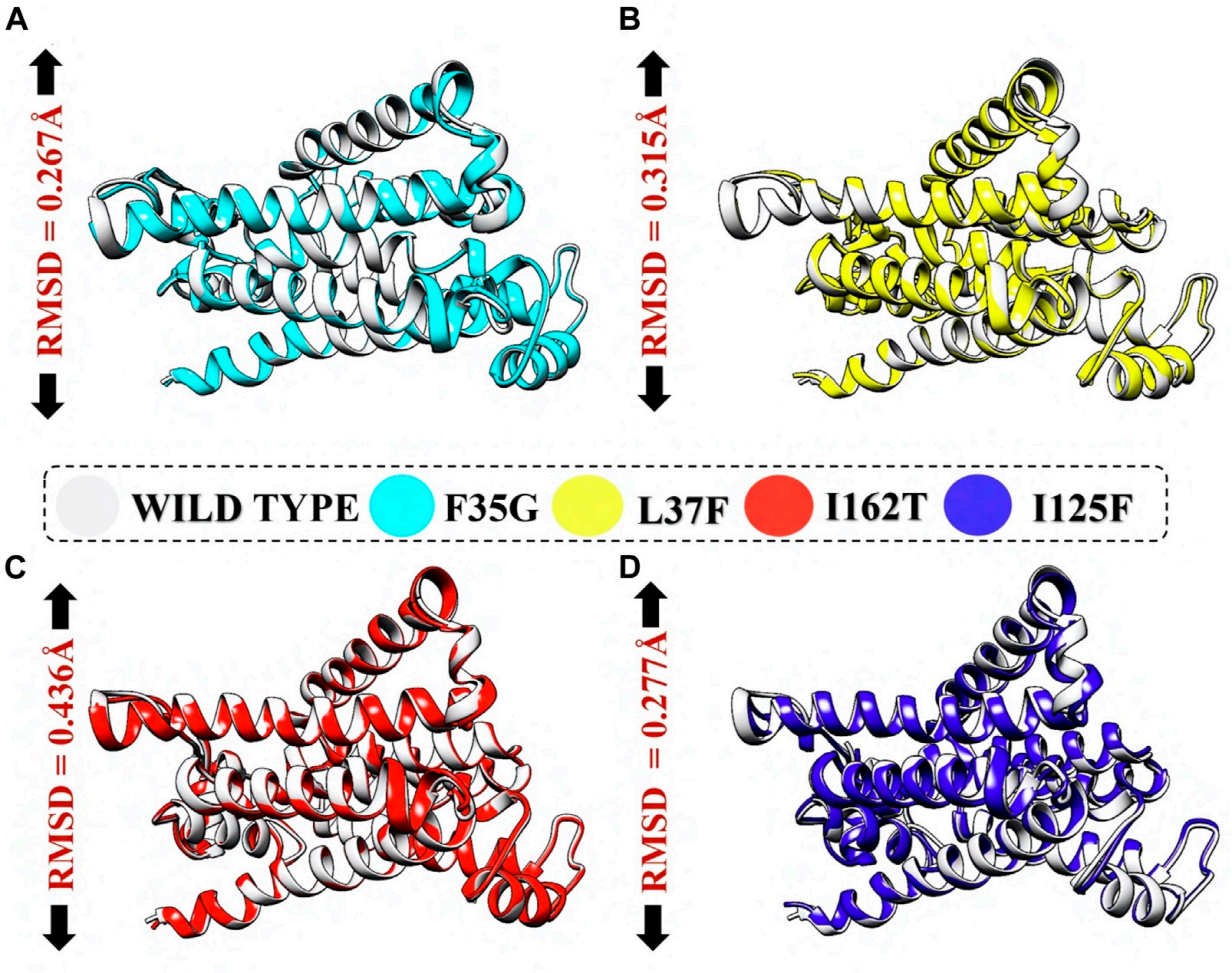
RMSD calculation by superimposing mutants on wildtype NSP6. **(A)** Represents deviation of F35G mutant. **(B)** Represents deviation of L37F mutant. **(C)** Represents deviation of I162T mutant. **(D)** Represents deviation of I125F mutant.

The structural variances between the wild-type ORF6 protein and the generated mutants were assessed by superimposing their respective structures, and the Root Mean Square Deviation (RMSD) values were determined. The calculated RMSD values (0.26 Å, 0.31 Å, 0.43 Å, and 0.27 Å) for the F35G, L37F, I162T, and I125F mutants indicated significant differences compared to the wild-type protein (Figures 4A–D). These mutations led to changes in the secondary structure and overall conformation of the protein, highlighting the need to investigate their potential impact on the binding affinity between NSP6 and TBK1. Consequently, structural procedures such as molecular docking, were utilized to find out the effect of these mutations on downstream immune escape mechanism.

### Molecular docking of wildtype and mutant NSP6 with TBK1

**FIGURE 5:**
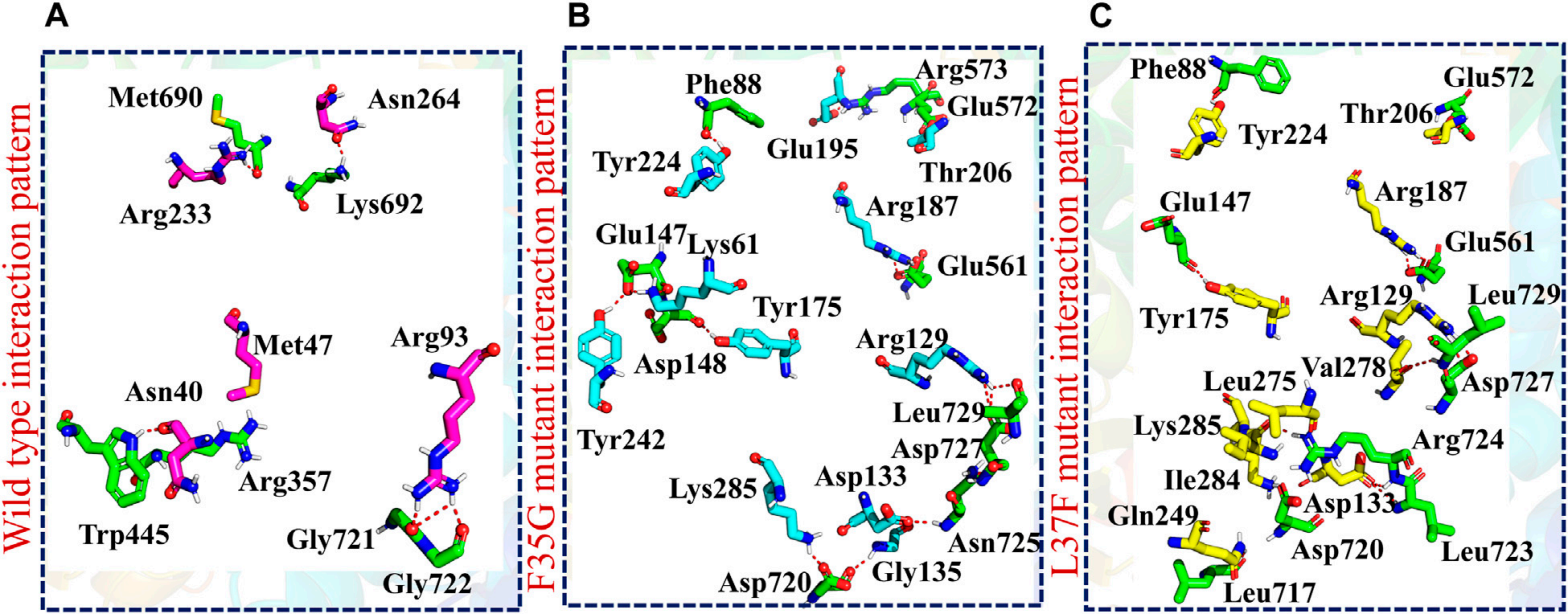
Molecular docking analysis of wildtype and mutant NSP6-TBK1 complexes. **(A)** Showing the binding residues of wildtype-TBK1 complex. **(B)** Showing the binding residues of F35G-TBK1 complex. **(C)** Showing the binding residues of L37F-TBK1 complex.

Molecular docking is a versatile and powerful tool for studying protein-protein interactions, providing valuable insights into biology, drug discovery, and personalized medicine. Its computational nature allows researchers to explore a wide range of interactions and structural conformations, complementing experimental approaches and guiding further studies. The function of NSP6, a non-structural protein of SARS-CoV-2, has been revealed to hinder the induction of IFNβ by interacting with Tank Binding Kinase 1 (TBK1), allowing the virus to evade the human immune system (Guo et al., 2021; Vazquez et al., 2021). Consequently, we used the molecular docking approach to check the effect of identified variants (F35G, L37F, L125F, and I162T) on the binding affinity of NSP6 and TBK1. The predicted docking score for the wildtype-TBK1 complex was recorded to be −1436.2. The binding interface analysis by PDBsum revealed the formation of 6 hydrogen bonds, 1 salt bridge, and 147 non-bonded contacts. The residues involved in the hydrogen bonds formation were Arg357-Met47, Gly722-Arg93, Gly721-Arg93, Lys692-Asn264, Trp445-Asn40 and Met690-Arg233 however, the salt bridge was formed between Glu696 and Lys63 (Figure 5A; Supplementary Figure S1A). Furthermore, the analysis of F35G-TBK1 complex by ClusPro server showed a docking score of −1723.2 while the interaction interface analysis revealed the formation of 15 hydrogen bonds, 7 salt bridges, and 250 non-bonded contacts. The key hotspot residues Asn725-Asp133, Asp720-Gly135, Asp720-Lys285, Asp727-Arg129, Leu729-Arg129, Asp148-Tyr175, Glu147-Lys61, Glu147-Tyr242, Phe88-Tyr224, Glu561-Arg187, Arg573-Glu195 and Glu572-Thr206 were involved in the hydrogen bonds formation (Figure 5B; Supplementary Figure S1B). The docking score of −1788.6 was recorded for the L37F-TBK1 complex however, the interface analysis showed the presence of 12 hydrogen bonds, 4 salt bridges, and 303 non-bonded contacts. The key amino acid residues Leu717-Gln249, Asp720-Lys285, Leu723-Asp133, Arg724-Asp133, Arg724-Ile284, Arg724-Leu275, Leu729-Val278, Asp727-Arg129, Glu147-Tyr175, Phe88-Tyr224, Glu561-Arg187 and Glu572-Thr206 formed the hydrogen bonds while, Asp720-Lys285, Arg724-Asp133, Asp727-Arg129 and Glu561-Arg187 formed the salt bridges (Figure 5C; Supplementary Figure S1C).

**FIGURE 6:**
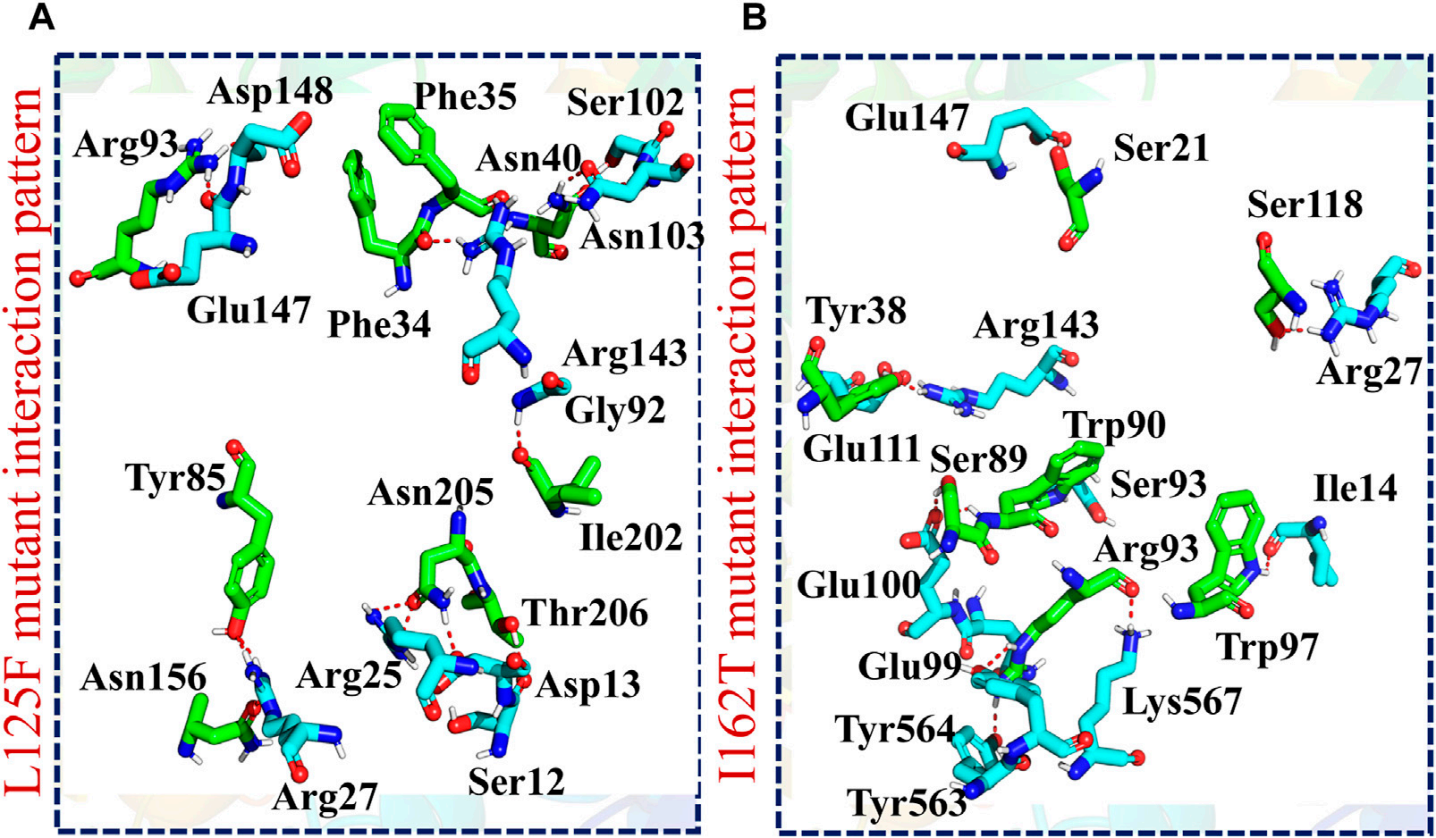
Molecular docking analysis of L125F and I162T-TBK1 complexes. **(A)** Showing the binding residues of L125F-TBK1 complex. **(B)** Showing the binding residues of I162T-TBK1 complex.

After this, the ClusPro server predicted the docking score of −1510.2 for the L125F-TBK1 complex. The PDBsum analysis showed the presence of 14 hydrogen bonds and 180 non-bonded contacts. The residues involved in hydrogen bonds formation were Asp148-Arg93, Glu147-Arg93, Arg143-Phe35, Arg143-Phe34, Gly92-Ile202, Arg27-Tyr85, Arg27-Asn156, Ser12-Thr206, Asp13-Asn205, Arg25-Asn205, Asn103-Asn40 and Ser102-Asn40 (Figure 6A; Supplementary Figure S1D). Finally, the analysis of I162T-TBK1 complex showed the docking score of −1551.7 however, the binding interface analysis revealed the existence of 13 hydrogen bonds, 1 salt bridge and 204 non-bonded contacts. The key residues Ile14-Trp97, Lys567-Arg93, Tyr563-Arg93, Glu99-Arg93, Tyr564-Arg93, Glu100-Ser89, Glu100-Trp90, Ser93-Trp90, Arg27-Ser118, Arg143-Tyr38, Glu111-Tyr38 and Glu147-Ser21 formed the hydrogen while Glu99-Arg93 formed the salt bridge in the I162T-TBK1 complex (Figure 6B; Supplementary Figure S1E). These results indicated that the shortlisted NSP6 mutants had significantly increased the binding affinity of NSP6 and TBK1 as compared to the wildtype complex. This strengthened interaction has raised concerns about the potential of SARS-CoV-2 to evade the human immune system. Among the mutants studied, the F35G variant demonstrated the highest binding affinity with TBK1, as evident from the docking score and hydrogen bonding network analysis. As a result, we chose to concentrate our further investigations on this specific mutant to explore its potential as a target for drug discovery and development to halt the interactions between NSP6 and TBK1.

### Binding free energies calculations

Binding free energy calculations are essential for elucidating the energetics and dynamics of protein-protein interactions. Binding free energy calculations serve as a validation tool for computational models predicting protein-protein interactions. Comparing calculated binding energies with experimental data helps assess the accuracy of the computational methods used (Khan et al., 2021; Khan et al., 2022a). Therefore, to validate our docking results, we used the MM/GBSA approach to evaluate the binding free energies of both wildtype and mutant NSP6-TBK1 complexes. According to Table 2, the van der Waals (vdW) energy for the wild-type NSP6-TBK1 complex was calculated to be −213.46 kcal/mol. Interestingly, the mutants F35G, L37F, L125F, and I162T showed significant increases in vdW energy, with values of −279.9 kcal/mol, −281.93 kcal/mol, −217.48 kcal/mol, and −249.71 kcal/mol, respectively. This suggests a notable variation in the NSP6 variants compared to the wild type, aligning with previous studies on different SARS-CoV-2 variants that also pointed to increased vdW energy. Another crucial factor, the electrostatic energy, was reported to be responsible for the enhanced binding of various variants in prior research (Suleman et al., 2021b; Shafiq et al., 2022; Suleman et al., 2023b). In the current study, similar observations were made as the wild type exhibited an electrostatic energy of −1059.82 kcal/mol. Conversely, the F35G, L37F, L125F, and I162T variants showed higher electrostatic energies, measuring −1342.8 kcal/mol, −1371.79 kcal/mol, −595.31 kcal/mol, and −647.11 kcal/mol, respectively. Regarding the overall binding energy, the wild-type NSP6-TBK1 complex displayed a value of −118.12 kcal/mol, whereas the F35G, L37F, L125F, and I162T mutants had binding energies of −172.19 kcal/mol, −149.05 kcal/mol, −122.96 kcal/mol, and −116.22 kcal/mol, respectively (Table 3). The outcomes strongly support the notion of the mutant F35G having the highest binding free energy, confirming the results obtained from molecular docking.

**TABLE 3:** Binding free energies of wildtype and mutant NSP6-TBK1 complexes.

Complexes	vdW	Electrostatic	GB	SA	Total binding energy
Wild Type	−213.46	−1059.82	1182.2	−27.04	−118.12
F35G	−279.9	−1342.8	1484.89	−34.38	−172.19
L37F	−281.93	−1371.79	1539.86	−35.19	−149.05
L125F	−217.48	−595.31	714.75	−24.91	−122.96
I162T	−249.71	−647.11	811.34	−30.74	−116.22

### Virtual drug screening against NSP6

In the field of drug design, virtual drug screening serves as a vital tool, enabling researchers to efficiently pinpoint and assess potential drug candidates prior to undertaking resource-intensive and time-consuming laboratory experiments. By offering a faster and more efficient approach, virtual drug screening plays a crucial role in identifying promising drug candidates and refining their chemical and biological properties during the drug design process (Maia et al., 2020; Oliveira et al., 2023). Before initiating the database screening, Lipinski’s rule of five was applied to filter out drug-like molecules. Among the total molecules in the database, 723 compounds successfully met the ADMET criteria. For virtual drug screening, AutoDock Vina was utilized, revealing docking scores ranging from −6.69 to −0.23 kcal/mol. Compounds with scores lower than −6.69 were shortlisted for further analysis. Among these, 38 compounds were subjected to induced fit docking, resulting in docking scores ranging from −6.7 to −3.6 kcal/mol. The top five hits were then selected based on their docking scores and hydrogen bonding network. The top five compounds, namely, 10-[(2Z)-3,7-dimethylocta-2,6-dienyl]-5, 9,11-Trihydroxy-3,3-dimethyl-pyrano[3,2-a]xanthen-12-one (C1), 6,11-dihydroxy-9,10-Dimethoxy-3, 3-dimethyl-5-(4-methylpent-3-enyl) Pyrano [2,3-c] xanthen-7 one(C2),1-(3-acetyl-2-Hydroxy-4,6-dimethoxy-phenyl)-4,5-Dihydroxy-2-methyl- anthracene 9,10-dione(C3), [(2R,3R)-2-(3,4-dihydroxyphenyl)-5,7-Dihydroxy-chroman-3-yl](C4), and (2S,3S)-2-(3,4-dihydroxyphenyl)-5,7-dihydroxy-3-[(2R,3R,4R,5S)-3,4,5Trihydroxytetrahydropyran-2-yl] (C5) with a docking score of −6.59 kcal/mol, −6.52 kcal/mol, −6.32 kcal/mol, −6.22 kcal/mol and −6.21 kcal/mol respectively were selected for further analysis. The top hits compounds along with their docking scores are shown in Table 4.

**TABLE 4:** List of top five hits, along with their 2D-structures and docking scores.

Top hit#	IDs	Drug name	2D-structure	Docking score
1	AN_UY_045_1	10-[(2Z)-3,7-dimethylocta-2, 6-dienyl]-5, 9,11-Trihydroxy-3, 3-dimethyl-pyrano[3,2-a] xanthen-12-one	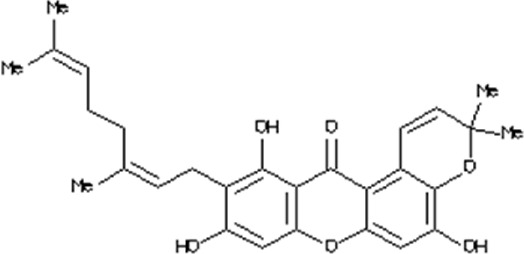	−6.59 kcal/mol
2	BMC_00066	6,11-dihydroxy-9,10dimethoxy-3,3-Dimethyl-5-(4-methylpent-3-enyl) Pyrano [2,3-c] xanthen-7-one	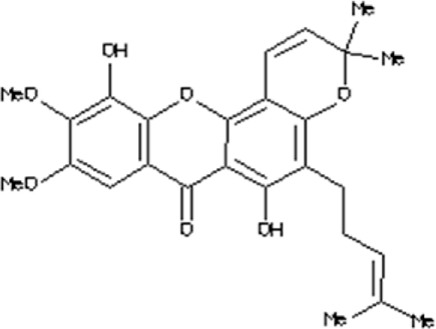	−6.52 kcal/mol
3	SA_0133	1-(3-acetyl-2-Hydroxy-4,6-dimethoxy-phenyl)-4,5-dihydroxy-2-methyl-anthracene-9, 10-dione	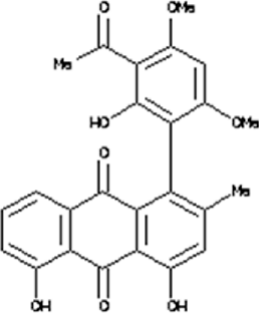	−6.32 kcal/mol
4	WA_0027	[(2R,3R)-2-(3,4- Dihydroxyphenyl)-5,7-dihydroxy-chroman-3-yl]	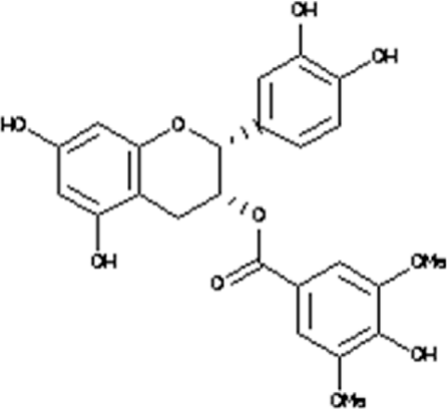	−6.22 kcal/mol
5	WA_0088	(2S,3S)-2-(3,4-Dihydroxyphenyl)-5,7-Dihydroxy-3-[(2R,3R,4R,5S)-3,4,5-trihydroxytetrahydropyran-2-yl]	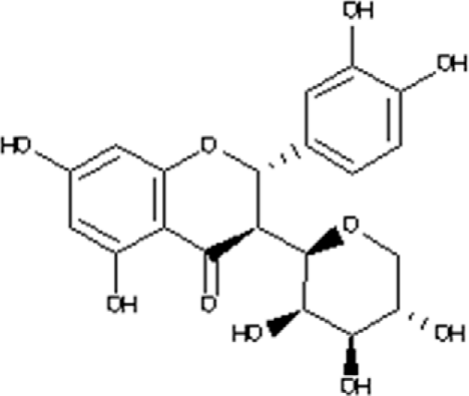	−6.21 kcal/mol

### Interaction analysis of top hit compounds

**FIGURE 7:**
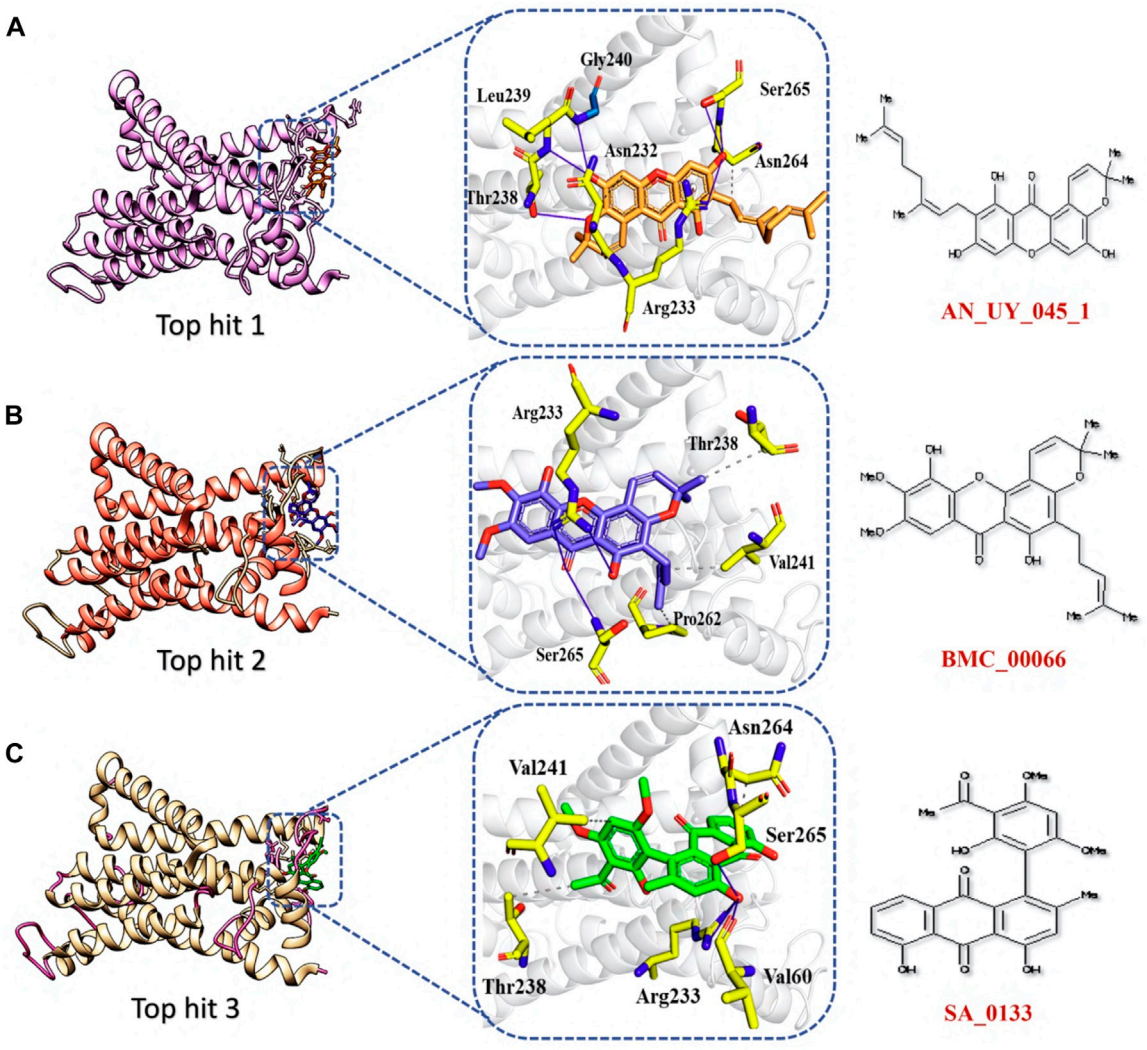
Binding interface analysis of top hit 1-3 and NSP6. **(A)** Represents the binding network between top hit 1 and NSP6. **(B)** Represents the binding network between top hit 2 and NSP6. **(C)** Represents the binding network between top hit 3 and NSP6.

The top 5 hits were analyzed in detail, focusing on the interactions involving hydrophobic interactions, hydrogen bonds, and salt bridges. The analysis of tophit1-NSP6 complex displayed a docking score of −6.59 kcal/mol. This complex formed six hydrogen bonds and one hydrophobic bond with specific residues within the target protein. The key residues involved in establishing this bonding network were Leu239, Thr238, Gly240, Asn232, Arg233, Ser265, and Asn264 (Figure 7A). Similarly, our analysis of the top hit 2 compound revealed that it establishes 3 hydrogen bonds and 3 hydrophobic interaction with specific amino acids in the target protein, including Arg233, Ser265, Pro262, Val241, and Thr238. The docking score for the top hit2 was recorded to be −6.52 kcal/mol (Figure 7B). Furthermore, the docking score of −6.32 kcal/mol was recorded for the top hit 3-NSP6 complex. The binding interface analysis showed the presence of 4 hydrogen bonds and 3 hydrophobic bonds with amino acids Val241, Thr238, Arg233, Val60, Ser265 and Asn264 (Figure 7C).

**FIGURE 8:**
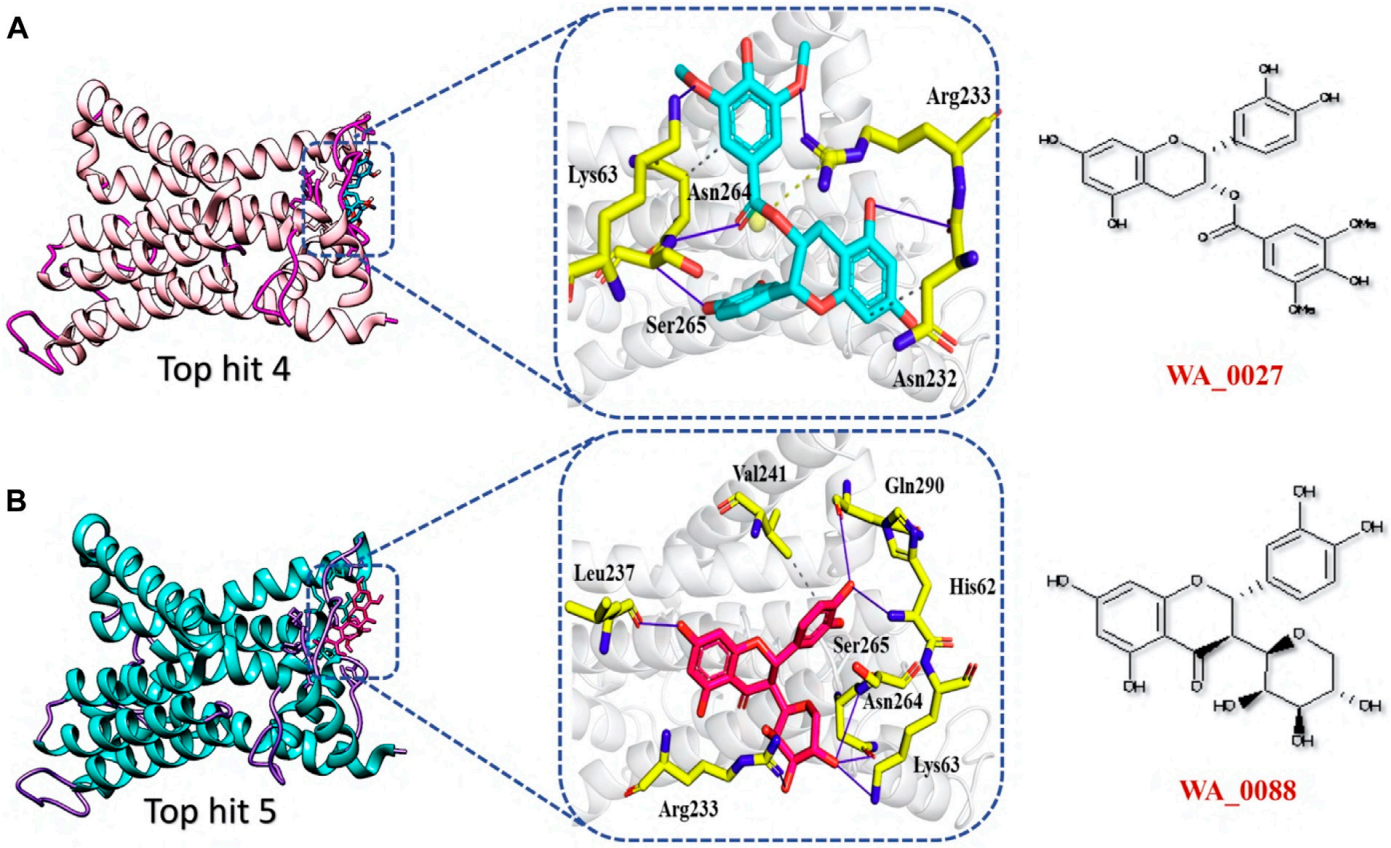
Binding interface analysis of top hit 4 and top hit 5-NSP6 complex **(A)** represents the binding network between top hit 4 and NSP6. **(B)** Represents the binding network between top hit 5 and NSP6.

The analysis of top hit 4-NSP6 complex showed the existence of 5 hydrogen bonds, 2 hydrophobic bonds, and 1 salt bridge with amino acids residues including, Lys63, Ser265, Asn264, Asn232, and Arg233. The docking score for the top hit 4-NSP6 complex was found to be −6.22 kcal/mol (Figure 8A). Similarly, in the case of the top hit 5-NSP6 complex, there were favorable interactions observed with the target protein. This complex formed 7 hydrogen bonds and 1 hydrophobic interaction, involving specific amino acids, namely, Leu237, Arg233, Lys63, Asn264, Ser265, His62, Gln290, and Val241. The docking score for this interaction was −6.21 kcal/mol (Figure 8B). The results of our study indicate that these compounds show great promise as potential drug candidates due to their favorable interactions with the target protein. These interactions are crucial for enhancing the compounds’ therapeutic efficacy, making them potentially effective treatments. To further verify the dynamic stability of durgs-NSP6 complex, we selected the top 3 hits for the molecular dynamic simulation analysis.

### Molecular dynamics simulations analysis of top hits

**FIGURE 9:**
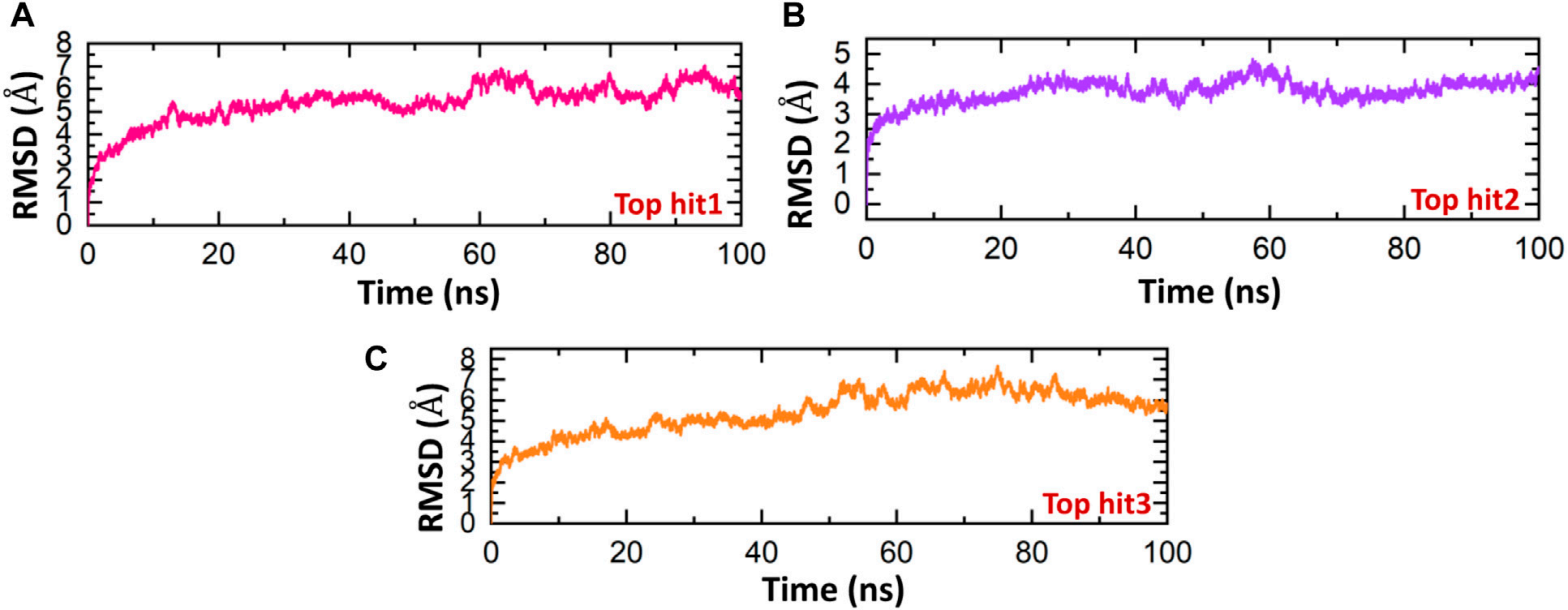
Dynamics stability analysis of drugs-NSP6 complex. **(A)** Showing the RMSD value of top hit 1 **(B)** showing the RMSD value of top hit 2. **(C)** Showing the RMSD value of top hit 3.

The stability of molecular interactions within a binding cavity is a critical factor in determining the binding affinity of a small molecular ligand. Simulation trajectories are commonly employed to analyze this stability, and one of the key metrics used is the RMSD. The RMSD provides valuable data on the dynamic stability of the interacting molecules, shedding light on the strength of their binding. Understanding a protein’s dynamic nature is crucial, as it aids in estimating the biological complex stability in a dynamic environment (Karplus and Kuriyan, 2005). In the current study we executed 100ns simulation to calculate the RMSD to check the stability of protein-drugs top hits in a dynamic environment. As shown in Figure 9, during the 100ns simulation no significant convergence was observed in the RMSD value of all the three top hits which indicates the stable behavior of protein-drug complexes. In case of the top hit 1, the system equilibrated at the 10ns and then remain stable until the end of simulation with the average RMSD of 5 Å (Figure 9A). However, in case of top hit 2 we reported the lowest RMSD (3 Å). The system equilibrated at the 3ns and remain significantly stable till 100 ns (Figure 9B). Finally, the top hit 3 system followed the similar pattern with the average RMSD of 5 Å. In case of the top hit 1 the system equilibrated at 3ns with the RMSD value of about 4 Å which raised steadily and reached 6 Å at 50 ns (Figure 9C).

**FIGURE 10:**
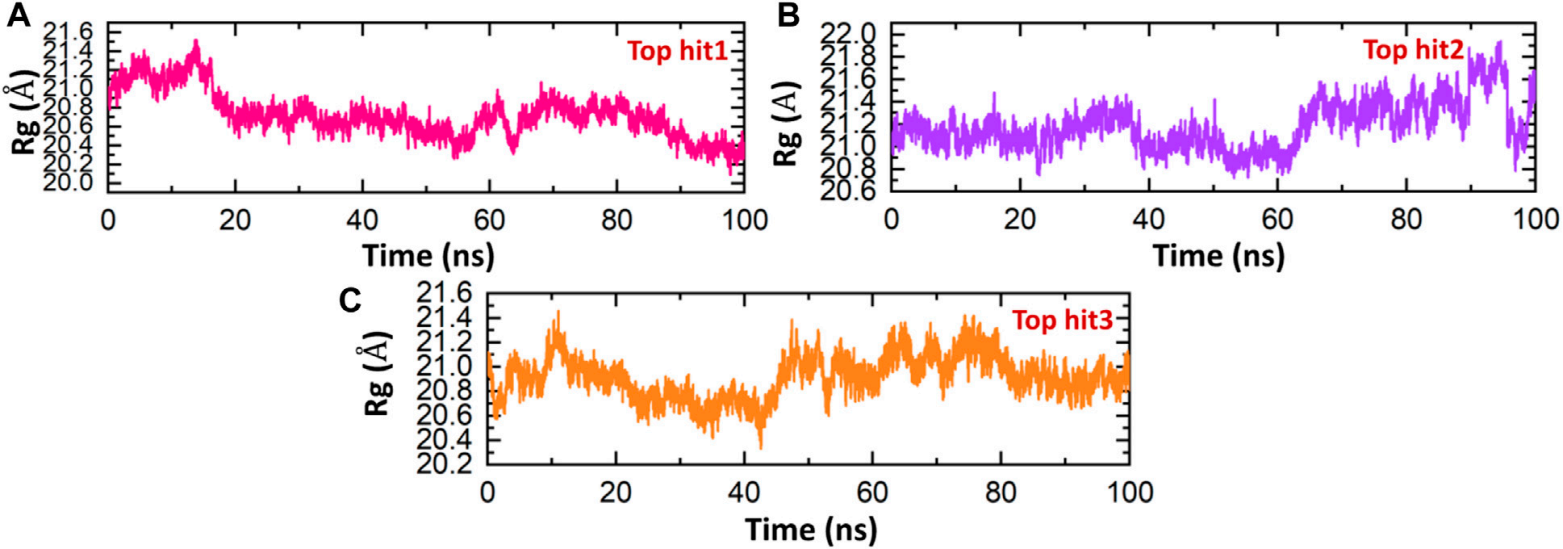
Compactness analysis of drug-NSP6 complex. **(A)** Showing the Rg value of top hit 1. **(B)** Showing the Rg value of top hit 2 **(C)** showing the Rg value of top hit 3.

The findings show that the top hits 1-3 exhibit consistent dynamics, indicating their stability, and have the potential to interact with the interface residue in a way that would effectively hinder the binding of NSP6 to TBK1. The stability of every complex was investigated in a dynamic environment to identify binding and unbinding events. This was achieved by monitoring the radius of gyration (Rg) over time, which served as a measure of the structural compactness. The degree of compactness of the protein complexes was found to be a crucial factor affecting their stability (Khan S. et al., 2022). The results depicted in Figure 10 exhibit a comparable trend in terms of compactness when compared to the RMSD. For top hit 1, the structure remained compact with an Rg value of 21 Å until 18 ns, after which it gradually decreased to 21.8 Å, although no substantial convergence was observed during the simulation time (Figure 10A). Similarly, for top hit 2, an average Rg value of 21.2 Å was recorded with some convergence observed between 70ns and 100 ns (Figure 10B). Lastly, top hit 3 followed a similar Rg pattern as top hit 2, but with a slightly lower Rg value of 20.8 Å (Figure 10C). The changes in Rg observed in the simulations are indicative of the binding and unbinding events between the ligands and the receptor. The observed Rg values suggest that top hits 1-3 exhibit stable binding to the receptor and hold potent pharmacological activity against NSP6.

**FIGURE 11:**
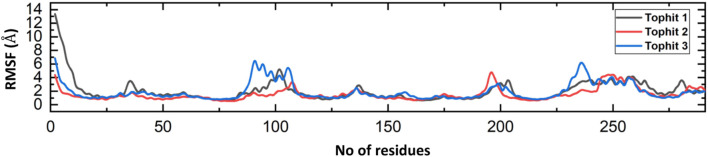
Fluctuation analysis of drug-NSP6 complexes at residues level.

Proteins are essential biomolecules that play crucial roles in various biological procedures in living organisms. Understanding the flexibility and rigidity of protein residues is crucial for comprehending these processes (Rashid et al., 2021; Shah et al., 2022). To investigate protein dynamics, scientists employ various methods, including the calculation of Root Mean Square Fluctuation (RMSF) of backbone C-alpha atoms. RMSF analysis provides insights into the degree of flexibility of different regions of a protein structure. In recent years, RMSF analysis has been widely utilized in numerous studies to elucidate the relationship between protein dynamics and function (Rashid et al., 2021; Khan et al., 2022c). In current study, RMSF was executed to assess the residual fluctuation of the top hit1-3 complexes. In Figure 11, it can be observed that most of the residues in the systems are in a state of equilibrium, with a mean RMSF of approximately 1 Å. The RMSF pattern for each complex is quite similar, with some fluctuations observed at specific amino acid residues such as 100, 200, and 240. A higher RMSF value at a certain residue suggests that the region is more flexible, while a lower value indicates minimal movement around its average position throughout the simulation. Importantly, the analysis of RMSF results indicates that the top three drugs show a higher binding affinity with the binding interface of human NSP6, as compared to other drugs.

**FIGURE 12:**
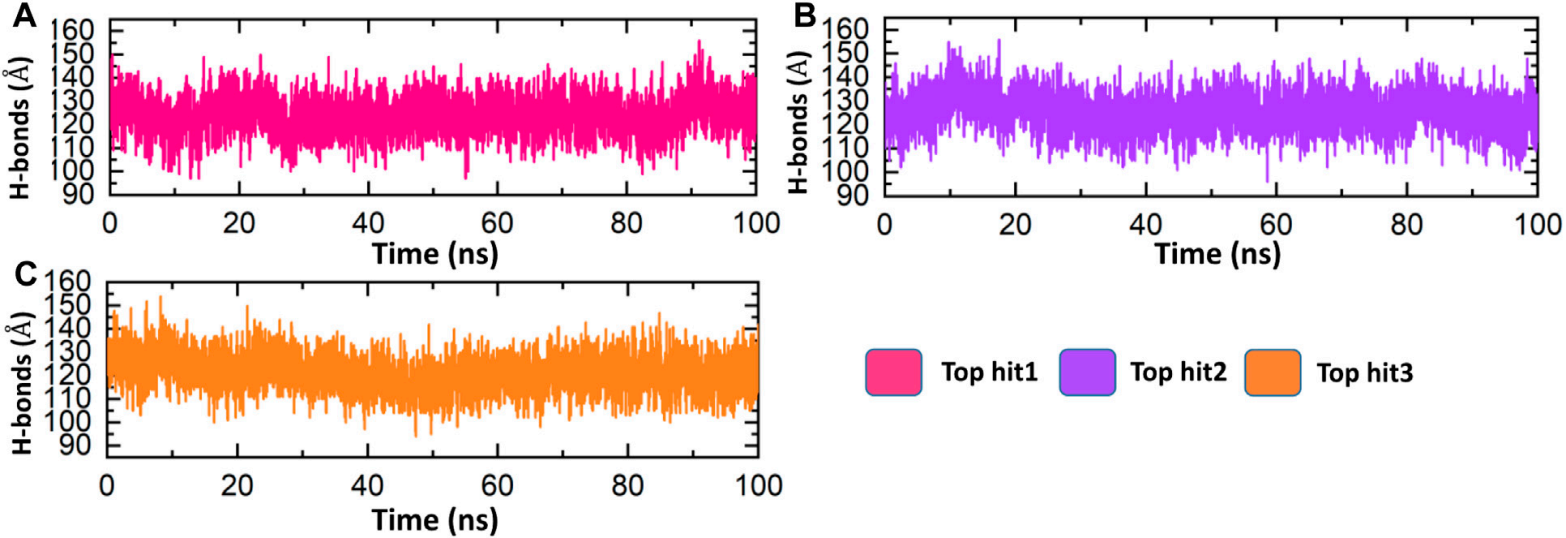
Bonding network analysis between top hit drugs and NSP6. **(A)** Showing the number of hydrogen bonds in top hit 1. **(B)** Showing the number of hydrogen bonds in top hit 2. **(C)** Showing the number of hydrogen bonds in top hit 3.

The evaluation of hydrogen bonds is a frequently used procedure for analyzing the binding efficiency among interacting molecules (Chen et al., 2016). It is essential to comprehend the patterns of hydrogen bonding involved in protein-drug interactions in order to accurately predict the strength of these interactions (Jewkes et al., 2011; Chodera and Mobley, 2013). To analyze the evolution of hydrogen bonding patterns during simulation, the number of hydrogen bonds was executed for each trajectory. The hydrogen bonding network for each complex was analyzed over time, and the findings are presented in Figure 12. As per Figure 12, it is obvious that all the complexes exhibit a robust hydrogen bonding network, indicating stable interactions among the identified lead drugs and NSP6. The hydrogen bonds formed between the top-ranked drug-NSP6 complexes (top hit 1-3), was found to be 130 (Figures 12A–C). These observations corroborate the outcomes obtained from molecular docking, RMSD, and RMSF analyses, providing additional evidence of the stability of the complexes.

## Conclusion

In this study, we identified 15 novel mutations in the NSP6 protein of SARS-CoV-2. Among these mutations, four (F35G, L37F, L125F, and I162T) were found to significantly destabilize the structure of NSP6. Furthermore, the molecular docking analysis revealed the highest binding affinity of mutant NSP6 and TBK1 as compared to wild type. Particularly, the F35G mutation exhibited the strongest binding affinity, supported by a calculated binding free energy of −172.19 kcal/mol. To disrupt the binding between NSP6 and TBK1, we conducted virtual drug screening to develop a novel inhibitor derived from natural products. From this screening, we identified the top 5 hit compounds as the most promising candidates, selected based on their bonding network and docking score. The molecular dynamic simulation further verified the dynamic stability of the top 3 hits-NSP6 complexes. However, it is essential to conduct experimental validation to confirm their efficacy. In conclusion, this study provides valuable insight into the higher infectivity of the SARS-CoV-2 new variants and a strong rationale for the development of novel drugs against NSP6.

## Data Availability

The original contributions presented in the study are included in the article/Supplementary Material, further inquiries can be directed to the corresponding authors.
