# Supplementary material for: Elucidating the binding mechanism of SARS-CoV-2 NSP6-TBK1 and structure-based designing of phytocompounds inhibitors for instigating the host immune response
Source: Front Chem. 2024 Jan 16;11:1346796. doi: 10.3389/fchem.2023.1346796 (PMC10824840; doi:10.3389/fchem.2023.1346796)
Supplement: Supplementary file 1 [file DataSheet1.docx]

**Supplementary figures**

**
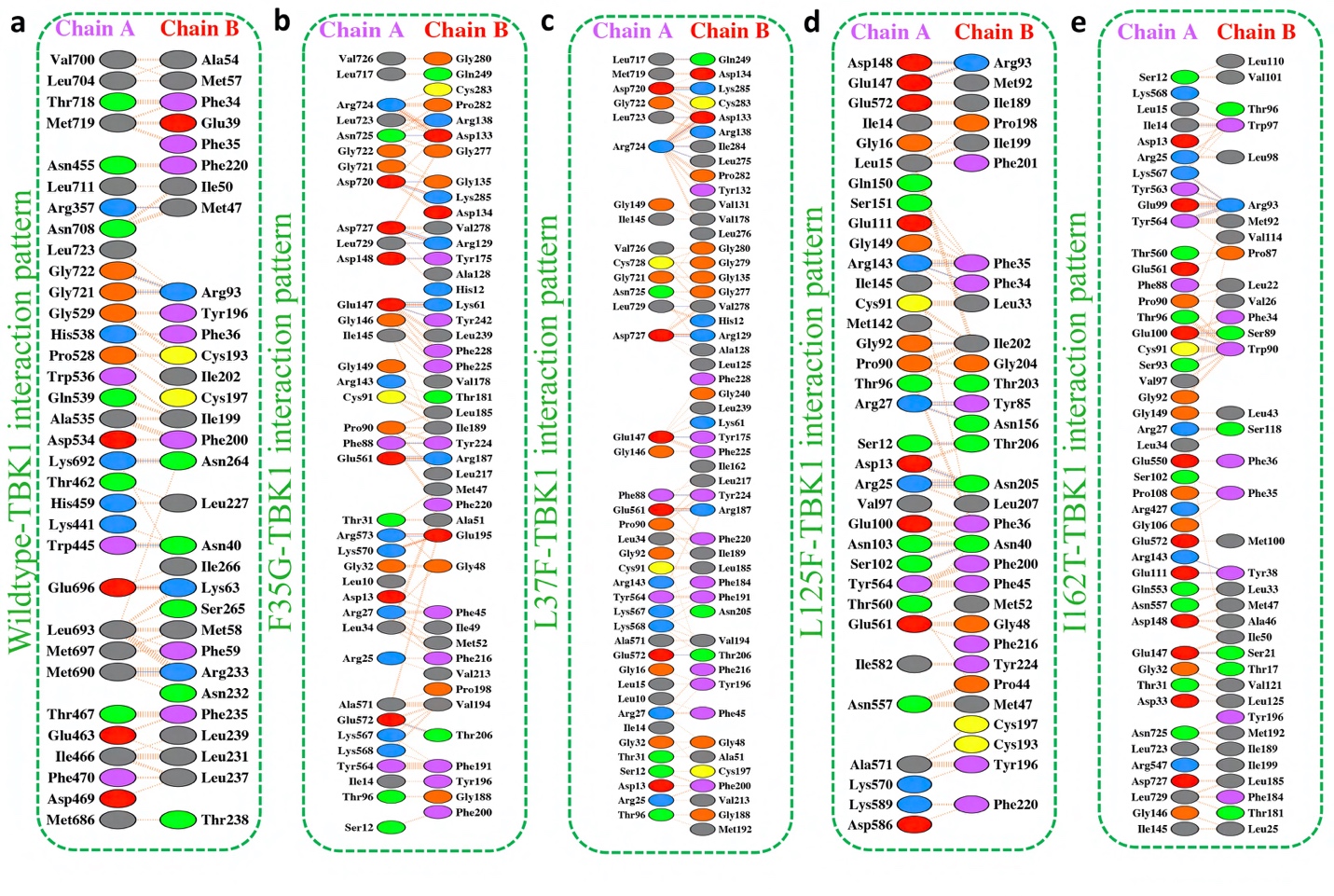
**

**Supplementary figure 1.** Interaction pattern of wildtype and mutant NSP6 and TBK1. (**a**) showing bonding network of wildtype NSP6 with TBK1. **(b)** showing bonding network of F35G with TBK1 (**c**) showing bonding network of L37F with TBK1. (**d**) showing bonding network of L125F with TBK1. (**e**) showing bonding network of I162T with TBK1.
